# Acute Myeloid Leukemia in Qatar (2010–2016): Clinical, Biological, and Prognostic Factors and Treatment Outcomes

**DOI:** 10.3389/fgene.2020.00553

**Published:** 2020-06-17

**Authors:** Halima El Omri, Ruba Yasin Taha, Adel Elomri, Nancy Kacem, Hesham Elsabah, Anil Yousaf Ellahie, Amna Gamil, Firyal Ibrahim, Dina Sameh Abdelrahman Soliman, Susanna Jane Lawson El Akiki, Zafar Nawaz, Ahmad Al Sabbagh, Abdelfatteh El Omri

**Affiliations:** ^1^Medical Oncology-Hematology Department, National Centre for Cancer Care and Research (NCCCR), Hamad Medical Corporation (HMC), Doha, Qatar; ^2^Division of Engineering Management and Decision Sciences, College of Science and Engineering, Hamad Bin Khalifa University, Doha, Qatar; ^3^Clinical Pharmacy, National Center for Cancer Care and Research, Hamad Medical Corporation, Doha, Qatar; ^4^Hematopathology Laboratory, Hamad Medical Corporation, Doha, Qatar; ^5^Cytogenetic and Molecular Laboratory, Hamad Medical Corporation, Doha, Qatar; ^6^Center of Excellence in Bionanoscience Research and Genomics and Biotechnology Section and Research Group, Department of Biological Sciences, Faculty of Science, King Abdulaziz University, Jeddah, Saudi Arabia

**Keywords:** acute myeloid leukemia, cancer epidemiology, cytogenetic, Qatar, remission, survival, WHO classification of AML

## Abstract

The current study retrospectively evaluated cytogenetic profiles, various prognostic factors, and survival outcomes in 128 acute myeloid leukemia (AML) patients (14 ≤ age ≤ 70 years) admitted to the National Center for Cancer Care and Research (NCCCR), Hamad Medical Corporation, Doha, Qatar, between January 2010 and December 2016. The median age at diagnosis was 43 years, and 80% were less than 60 years old; 75% of patients were male. Cytogenetic analysis was integrated into the World Health Organization 2008 classification and showed that the percentages of normal and abnormal karyotypes were similar, accounting for 48.4% of each group of patients. The AML risk stratification based on cytogenetic analysis resulted in the following distribution: 18% in the favorable risk group, 57% in the intermediate-risk group, 24% in the unfavorable risk group, and 1% unknown. Only 88 patients received therapy with curative intent; 67% achieved complete remission, increasing to 81% after inductions 1 and 2. The median overall survival (OS) and disease-free survival (DFS) in AML patients were 26.6 and 19.5 months, respectively. The 3-year OS and DFS were 40 and 36%, respectively. Prognostic factors including age, gender, white blood cell count, and risk stratification were not significantly associated with treatment outcomes, whereas response to treatment vs. failure was significantly associated with the outcome (*p* = 0.01). The current study supports the importance of cytogenetics as a useful tool in diagnosis, prognosis, and risk assessment in AML treatment.

## Introduction

Abnormal growth of white blood cells (WBCs) in the bone marrow, typically known as acute myeloid leukemia (AML), usually hampers normal blood cell production. Intensity of AML is higher in the white population (3.8 per 100,000) than in Asian people (3.2 per 100,000) (Howlader et al., 2012). AML is a common type of blood cancer, accounting for 80% of all leukemias. Males are more likely to be affected than females. The risk of AML increases with age, particularly in those above 60 years old. In the United States, the incidence of AML increases by around 10 times in patients above 65 years old, with a rate of 12.2 per 100,000 people (De Kouchkovsky and Abdul-Hay, 2016).

Acute myeloid leukemia is a heterogeneous group of diseases with several morphological, immunophenotypic, cytogenetic, and molecular genetic features (Döhner et al., 2010). Cytogenetic examination is considered the most important prognostic factor to predict clinical outcomes in AML patients (Döhner et al., 2010; De Kouchkovsky and Abdul-Hay, 2016). Cytogenetic results have been integrated into the World Health Organization (WHO) classification of AML. Three important multicenter clinical trials conducted by Cancer and Leukemia Group B (Byrd et al., 2002), the United Kingdom Medical Research Council (MRC) (Grimwade et al., 1998), and the Southwest Oncology Group (SWOG) (Slovak et al., 2000) demonstrated the importance of cytogenetic analysis and its significant impact on AML patients outcomes, leading to the stratification of AML risk into three groups: favorable, intermediate, and unfavorable.

The favorable risk group included balanced translocation t(8;21), t(15;17), inversion inv(16), and t(16;16); the intermediate risk group included normal karyotype (CN-AML), t(9;11), −Y (loss of the Y chromosome), +8 (trisomy of chromosome 8), +11, +13, +21, del(7q) (removal of the long arm of chromosome 7), del(9q), and del(20q); and the unfavorable risk group included complex karyotype, inv(3) or t(3;3), t(6;9), t(6;11), t(11;19), del(5q), −5 (monosomy of chromosome 5), and −7 (Grimwade et al., 1998; Slovak et al., 2000; Byrd et al., 2002; Marchesi et al., 2011). In the favorable risk group, the presence of additional chromosomal abnormalities has no significant effect on prognosis (Appelbaum et al., 2006).

An appropriate and accurate assessment of prognosis is fundamental to the management of AML. This involves stratifying patients according to their risk of treatment resistance or treatment-related mortality. Several prognostic factors, including cytogenetic analysis (Grimwade et al., 2010), age (Appelbaum et al., 2006), WBC count, *de novo* or secondary AML, presence of any antecedent hematological disease (Grimwade et al., 1998; Slovak et al., 2000), and performance status are used by physicians to choose the best treatment procedure—standard or increased treatment intensity, consolidated chemotherapy or allogeneic hematopoietic stem cell transplant (HSCT) (Byrd et al., 2002)—or, more crucially, to choose between established and investigational therapies (Grimwade et al., 1998, 2010; Slovak et al., 2000; Byrd et al., 2002; Appelbaum et al., 2006). High cytogenic risk AML patients are potential candidates for allogeneic HSCT, whereas low cytogenetic risk patients are candidates for intensive chemotherapy. In intermediate-risk AML, the most suitable treatment remains to be defined (Grimwade et al., 1998; Byrd et al., 2002; Döhner et al., 2010).

In newly identified AML patients with abnormal karyotype, cytogenetic analysis is also recommended for documenting complete remission (CR) (Grimwade et al., 1998; Slovak et al., 2000; Marcucci et al., 2004; Hirsch et al., 2014). Several studies have found that persistence of cytogenetic abnormalities found in leukemic blast cells at diagnosis, following chemotherapy induction, may predict a high relapse rate of leukemia and a poorer clinical outcome with lower disease-free survival (DFS) and overall survival (OS) rates (Grimwade et al., 1998, 2010; Slovak et al., 2000; Marcucci et al., 2004; Hirsch et al., 2014).

Despite many advances in diagnosis, prognosis and risk stratification, and treatment of AML, the cure rate remains modest, at 60–80% at first induction in young adult patients (age ≤60 years) and 30–40% in older individuals (Appelbaum et al., 2006; Odenike et al., 2011).

This study aimed to determine the cytogenetic profile of AML in adults, to correlate cytogenetic abnormalities to the WHO 2008 classification, to evaluate the risk stratification, and to study the response to treatment of AML patients in Qatar from 2010 to 2016.

## Materials and Methods

The current study was an observational investigation that was conducted retrospectively based on AML patients’ records, including those aged less than 70 years and more than 14 years, diagnosed and treated at the National Center for Cancer Care and Research (NCCCR), Hamad Medical Corporation, Doha, Qatar, between January 2010 and December 2016, in relation to WHO 2008 guidelines. The follow-up was a minimum 2 years from inclusion so the results were considered until 2018 onward. The study was approved by the Medical Research Center Institutional Review Board (MRC-IRB) for the research proposal number “17287/17, 15/5/20”, and was exempted from ethical approval.

### Patients

Among 208 patients diagnosed with AML in the department of clinical hematology at NCCCR, only 128 were included in this study after the exclusion of patients over 70 years and those with acute promyelocytic leukemia. Patient data regarding sex, age, nationality, hematological features, diagnosis date, WHO classification, cytogenetic abnormalities, risk stratification, first and further line of treatment, response to treatment, consolidation, date of relapse, bone marrow transplantation, date of last follow-up, and date and cause of death were collected (Döhner et al., 2010).

### Morphologic Evaluation

Peripheral blood smears and bone marrow aspirations were stained with Wright’s stain. Differential counts of at least 100 cells in the peripheral blood smear and of at least 500 cells in the bone marrow smear were performed. AML was defined by the presence of at least 20% blasts in bone marrow and/or peripheral blood samples, except for AML with t(15;17), t(8;21), inv(16), or t(16;16), and some cases of erythroleukemia. AML was classified according to the 2008 WHO classification (Döhner et al., 2010).

### Immunophenotyping

Immunophenotyping was performed using multicolor flow cytometry on bone marrow aspirate/peripheral blood using a CD45-gating strategy to identify the immunophenotype of the blasts. An acute leukemia panel of 28 antibodies in a four-color combination (FITC/PE/ECD/PC5 fluorescent conjugates) was used: (1) CD34/CD117/CD45/CD19, (2) CD14/CD13/CD45/CD64, (3) HLADR/CD7/CD45/CD5, (4) CD34/CD33/CD45/CD56, (5) CD19/CD10/CD45/CD3, (6) CD15/CD33/CD45/CD2, (7) CD9/CD19/CD45/CD4, (8) CD20/CD10/CD19/CD45, (9) cMPO/cCD79a/cCD3/sCD45, (10) TdT/sCD19/sCD3/sCD45, (11) CD36/CD11c/CD45/CD11b, and (12) CD41/glycophorin A/CD45/CD61(PC7).

Data acquisition and analysis were performed using a Navios flow cytometer (Beckman Coulter) and Novio software.

### Cytogenetic Analysis

Cytogenetic analysis was performed in bone marrow or blood cells during metaphase. Karyotypes were identified following the rules of the International System for Human Cytogenetic Nomenclature (Gonzalez Garcia and Meza-Espinoza, 2006). Clonal abnormalities were considered when at least two metaphases showed the same aberration either in the structure or in the extra chromosome. Monosomy was considered significant if a minimum of three metaphases showed the same abnormality. Cytogenetic risk groups were assessed using the SWOG/ECOG (Southwest Oncology Group/Eastern Cooperative Oncology Group) criteria (Slovak et al., 2000). Fluorescence *in situ* hybridization (FISH) analysis was performed using specific probes for inv(16)(p13;q22), t(15;17)(q22;q21), t(8;21)(q22;q22), and 11q2.3 abnormalities, for mixed lineage leukemia (MLL) involving translocations, and for abnormalities (deletions or trisomy) of chromosome 5, 7, 8, 9, 11, 13, or Y. For all FISH analyses, at least 200 interphase nuclei were examined.

### Molecular Mutation Analyses

Gene mutation analyses were performed for *FLT3*-ITD, *NPM1*, and c-*Kit*.

### Treatment

Treatments were administered based on NCCCR’s AML guidelines. Treatment in patients 14 ≤ age ≤ 70 years old consists of double induction therapy and consolidation therapy based on cytogenetic stratification (high-dose cytarabine and/or allogeneic transplant). The induction chemotherapy (3 + 7) regimen consists of standard-dose cytarabine (200 mg/m^2^/d) continuous intravenous (IV) infusion on days 1–7 and anthracycline (idarubicin 12 mg/m^2^/d or daunorubicin 60 mg/m^2^/d or mitoxantrone 10–12 mg/m^2^/d IV on days 1–3). Consolidation consists of high-dose cytarabine (3 g/m^2^) IV every 12 h on days 1, 3, and 5. Patients aged between 60 and 70 years old, with a good performance status (less than 2), no comorbidities, and no adverse cytogenetics are treated with one induction (3 + 7 regimen) followed by 2–3 consolidations with intermediate-dose cytarabine (1000–1500 mg/m^2^) IV over 3 h every 12 h on days 1, 3, and 5. Finally, non-fit patients and those older than 70 years are treated with low-dose cytarabine (20–40 mg subcutaneously on days 1–10 for 4–5 weeks) or with hypomethylating agents (azacitidine 75 mg/m^2^ subcutaneously on days 1–7 or days 1–5, 8, and 9 every 28 days until progression).

### Statistical Analysis

Data were analyzed using SPSS statistical software 23.0. Differences in proportions were evaluated by Chi-square test. A value of *p* < 0.05 was considered to indicate a statistically significant difference. OS was determined based on the time between diagnosis and death or the time of the final clinical evaluation. DFS was defined as the time from CR to relapse or death or last follow-up. The Kaplan–Meier method was used to estimate OS and DFS, and survival curves were compared using the log-rank test. Cox proportional regression was used for the multivariate analysis. Odds ratios were calculated and reported with 95% confidence intervals. CR was confirmed when all the following conditions were fulfilled: less than 5% of blasts in the bone marrow, no leukemic blasts in the peripheral blood or extramedullary sites, and recovery of blood counts.

## Results

### Demographic Characteristics of AML Patients

**FIGURE 1:**
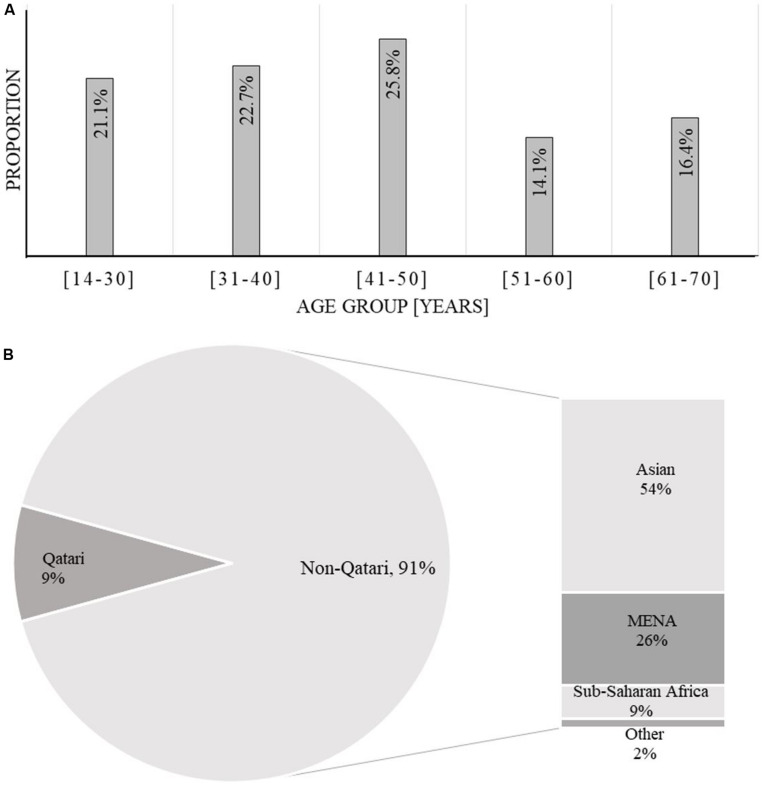
Cohort demographics by age **(A)** and origin **(B)**. Gender ratio (M/F) = 2.76.

Of the 128 AML patients diagnosed and treated at NCCCR, Qatar, from January 2010 to December 2016, 97 (76%) were male and 31 (24%) were female, constituting a male to female ratio of 3.12:0.32. AML was more common in males than females in this sample, consistent with previous estimates (Howlader et al., 2012). Patient age ranged between 14 and 70 years with a median of 43.3 years; 103 patients, accounting for 80%, were younger than 60 years old (Table 1 and Figure 1). Qatari patients (*n* = 11) represented 8.5% of the total, while non-Qataris (*n* = 117) represented 91.5%; the latter were mainly from South Asia (36%) and the Middle East/North Africa region (26%) (Table 1 and Figure 1).

**TABLE 1:** Demographics and presentation of AML by age, gender, and origin.

	**Age group**					**Total (%)**
	**14–30 year**	**31–40 year**	**41–50 year**	**51–60 year**	**61–70 year**	
Number of patients	27 (21)	29 (23)	33 (26)	18 (14)	21(16)	128 (100)
Sex, male, no. (%)	19 (70)	22 (76)	25 (76)	13 (72)	15(71)	94 (73)
Region, total, male, no. (%)						
Qatari	4, 3 (75)	1, 0 (0)	1, 0 (0)	2, 1 (50)	3, 1(50)	11 (9)
**Non-Qatari**						
East Asia and Pacific	5, 0 (0)	5, 3 (60)	6, 5 (83)	6, 5 (83)	1, 1(100)	23 (18)
Europe and Central Asia					1, 1(100)	1 (1)
Middle East and North Africa	7, 6 (86)	8, 6 (75)	7, 3 (43)	5, 4 (80)	6, 2(33)	33 (26)
North America			1, 1 (100)		1, 1(100)	2 (2)
South Asia	10, 9 (90)	13, 12 (92)	14, 14 (100)	4, 3 (75)	5, 5(100)	46 (36)
Sub-Saharan Africa	1, 1 (100)	2, 1 (50)	4, 2 (50)	1, 0 (0)	4, 4(100)	12 (9)

*All percentages are rounded to the nearest integer.*

### Hematologic Characteristics of AML Patients

At the time of diagnosis, WBC ranged between 0.7 and 307 × 10^3^/mm^3^, with a median of 20 × 10^3^/mm^3^. Hemoglobin distribution was in a range of 2.9–14.9 g/dL with a median of 8.1 g/dL. The platelet count median was 54.5 × 10^3^/mm^3^ in the range 3–1306. The median peripheral blast was 40% in the range 0–70, and the median marrow blast percentage was 54%, distributed between 20 and 99% (Table 2).

**TABLE 2:** Hematologic values: median and range.

	**Median**	**Range**
White blood cell count (10^3^/mm^3^)	20	(0.7–307)
Platelet count (10^3^/mm^3^)	54.5	(3–1306)
Hemoglobin count (g/dL)	5.4	(2.9–14.9)
Peripheral blasts (%)	40	(0–70)
Marrow blasts (%)	54	(20–99)

### Cytogenetics and Molecular Analysis of AML Patients

Karyotype analysis was performed in all patients and was considered to have failed in four patients (3.1%) because of inadequate metaphases. CN-AML and abnormal karyotype were each observed in 62 patients, accounting for 48.4% of each group of patients (Table 3). Molecular analysis was performed for 16 patients, focusing on *FLT3*-ITD and *NPM1* mutations. Mutant *FLT3*-ITD/mutant *NPM1* was found in one case, mutant *FLT3*-ITD/wild-type *NPM1* in five cases, wild-type *FLT3*-ITD/mutant *NPM1* in four cases, and wild-type *FLT3*-ITD/wild-type *NPM1* in six cases. The cohort’s results according to the WHO 2008 AML classification are summarized in Table 4.

**TABLE 3:** Cytogenetic results.

Number of patients	128 (100)
**Karyotype, no. (%)**	
Normal	62 (48.4)
**Abnormal**	
t(8,21)(q22;q22)	11 (8.6)
inv(16)(p13;q22) \t(16,16)(p13, Q22)	10 (7.8)
11q23	5 (3.9)
t(9;11)(p22;q23)	3 (2.3)
t(6;9) (p22;q23)	1 (0.8)
inv. (3) (q21q26.2) or t(3;3) (q21;q26.2)	1 (0.8)
“−7” or del(7)	3 (2.3)
“+8”	7 (5.5)
“+21”	1 (0.8)
Complex	4 (3.12)
Other	18 (14)
Failed	4 (3.1)

**TABLE 4:** Acute myeloid leukemia (AML) results in accordance with WHO 2008.

**AML category as per WHO 2008 classification**	***n* (%)**
**Acute myeloid leukemia with recurrent genetic abnormalities**	
AML with t(8;21)(q22,q22); RUNX1-RUNX1T1	11 (8.6)
AML with inv(16)(p13,1q22) or t(16;16)(p13.1;q22); CBFB-MYH11	10 (7.8)
AML with t(9;11)(p22;q22); MLL T3-MLL	3 (2.3)
AML with (6;9) (p22;q23)	1 (0.8)
AML with inv(3) (q21q26.2) or t(3;3) (q21;q26.2)	1 (0.8)
Provisional entity: AML with mutated *NPM1*	4 (3.1)
AML with myelodysplasia-related changes	18 (14.1)
Therapy-related myeloid neoplasms	3 (2.3)
**Acute myeloid leukemia, not otherwise specified**	
Acute erythroid leukemia	1 (0.8)
Acute megakaryoblastic leukemia	1 (0.8)
Acute monoblastic/monocytic leukemia	14 (10.9)
Acute myelomonocytic leukemia	17 (13.3)
Acute panmyelosis with myelofibrosis	–
AML without maturation	27 (21.1)
AML with maturation	10 (7.8)
AML with minimal differentiation	7 (5.5)
Total	128 (100)

### Risk Stratification With SWOG/ECOG Criteria

Using the SWOG pretreatment risk criteria system, patients were divided into four groups: 73 patients representing the majority (57%) were in the intermediate group, 23 patients (18%) were in the favorable risk group, 31 patients (24%) were in the unfavorable risk group, and one patient (0.8%) was classified as unknown.

### Treatment and Outcomes

#### Main Outcomes

**FIGURE 2:**
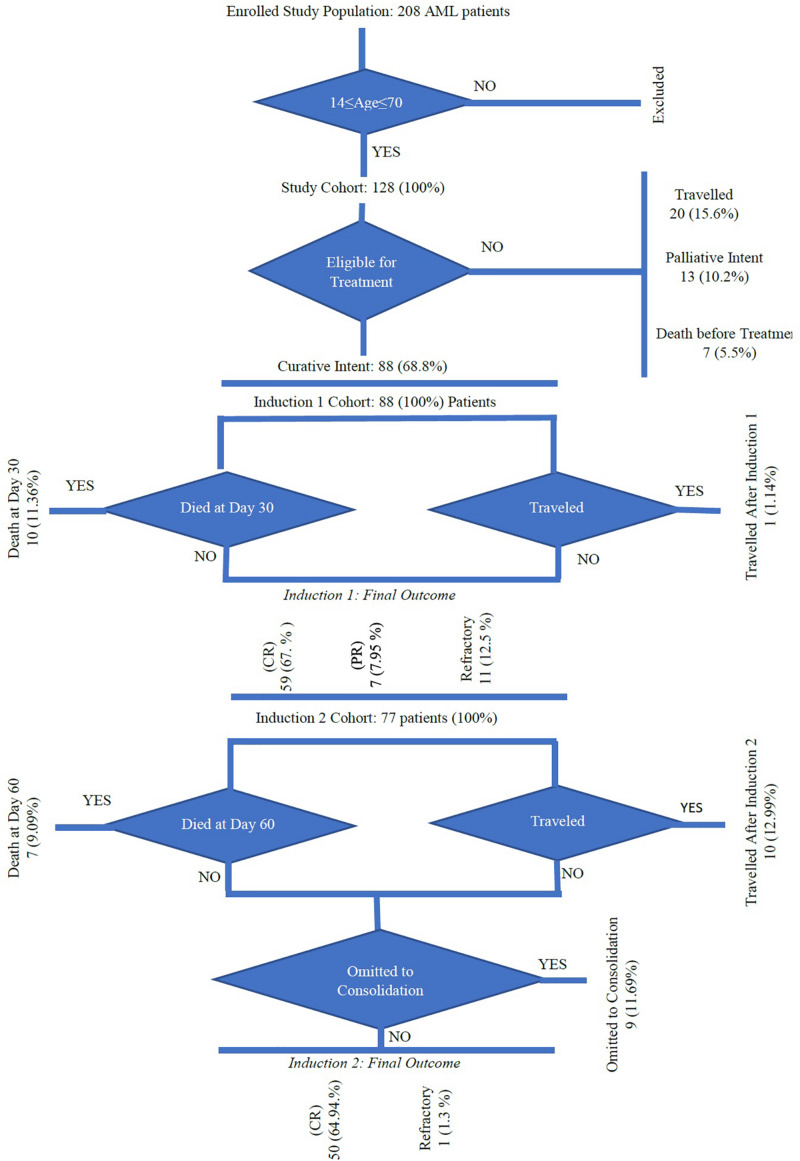
Plan of care summary.

Of the 128 patients, 88 (68.8%) received curative-intent treatment, 20 patients (15.6%) traveled back to their countries, 13 patients (10.2%) received palliative treatment, and seven patients (5.5%) died before treatment (Figure 2 and Supplementary Material). Fifty-nine patients (67%) were in CR, partial remission in 7 cases (7.95%), and refractory disease was present in 11 patients (12.5%). After the first induction (at day 30), 10 (11.3%) patients died and one patient traveled before evaluation. Seventy-seven patients received the second induction and/or salvage therapy; among them, CR was achieved in 50 (81%) of cases and 7 (9%) patients died by day 60. Ten (12.9%) patients traveled and another nine did not receive the second induction.

#### Cause of Death

Following the first and second inductions, a total of 17 patients died with microbial infection in 15 cases (88.2%) and cerebral bleeding in 2 cases (11.8%). The 15 deaths caused by microbial infection are summarized as follows: (1) Gram-negative bacilli (GNB) septicemia in eight cases caused by *Pseudomonas aeruginosa* and *Stenotrophomonas maltophilia* in two cases each; *Acinetobacter baumannii*, *Klebsiella oxytoca*, *Escherichia coli*, and *Burkholderia cepacia* in the other four cases, (2) Gram-positive cocci (GPC) septicemia in two cases caused by *Staphylococcus aureus* and *Enterococcus faecium*, and (3) invasive fungal infection in five cases caused by *Candida glabrata* in two cases, and *Candida krusei*, *Candida tropicalis*, and *Trichosporon asahii* in the remaining three cases.

#### Post-induction Therapy

Cytarabine was given to 67 patients in a larger dose (one cycle in 13 cases, two cycles in 21 cases, and 3 cycles in 18 cases). Allogeneic HSCT was administered to 19 patients (15 patients following the first CR and 4 following the second CR) (Supplementary Material).

#### Relapse

Two patients relapsed after chemotherapy and were alive in complete remission 2 (CR-2) following salvage therapy and allogeneic HSCT. Four patients relapsed after allogeneic HSCT and died (Supplementary Material).

**FIGURE 3:**
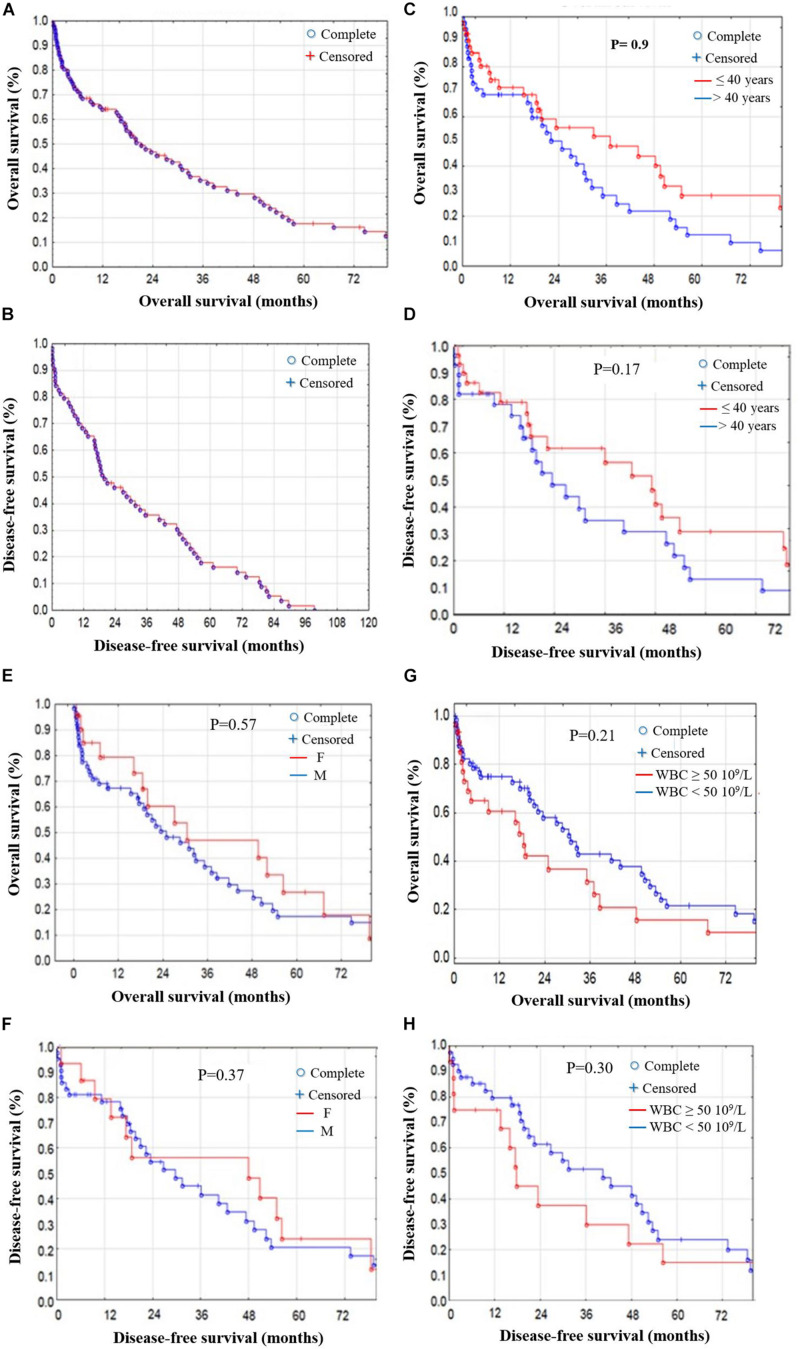
OS and DFS stratified by prognostic factors in AML patients. **(A)** OS and **(B)** DFS stratified in AML patients. **(C)** OS and **(D)** DFS stratified by age; no significant difference in OS (*p* = 0.9) or DFS (*p* = 0.17) for patients <40 or >40 years old. **(E)** OS and **(F)** DFS stratified by gender; no significant difference in OS (*p* = 0.57) or DFS (*p* = 0.37) between male and female patients. **(G)** OS and **(H)** DFS stratified by WBC findings; threshold values (≥50 × 10^9^/L) vs. (<50 × 10^9^/L) showed no significant difference in OS (*p* = 0.21) or DFS (*p* = 0.30).

**FIGURE 4:**
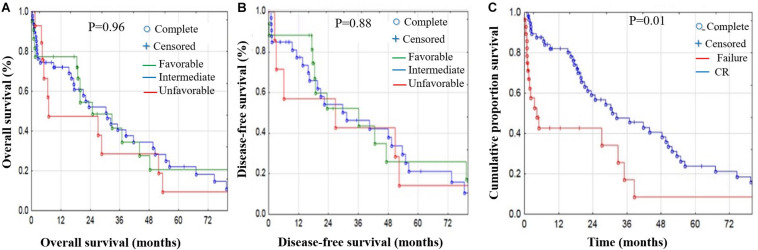
OS and DFS stratified by risk factors in AML patients. Risk stratification effect on OS **(A)** and DFS **(B)** showed no significant difference in OS or DFS; however, the unfavorable group showed poorer OS and DFS. **(C)** Cumulative proportion for treatment response-related survival; the CR patient group had significantly better OS and DFS compared with the non-CR group (*p* < 1%).

The median OS for the 88 patients who received curative-intent treatment was around 26.6 months, and the median DFS was about 19.5 months (Figures 3, 4). Prognostic factors including age, gender, WBC, risk stratification, and response to treatment showed no significant differences for OS and DFS (Figures 3A,B). We compared the age effect below and above 40 years old. Our results showed no significant difference for either OS (*p* = 0.9) or DFS (*p* = 0.17). In the current cohort study, the young patient group (<40 years of age) are presenting better OS and DFS than older patients. However, the difference was not significant enough to consider age as a risk factor (Figures 3C,D). Similarly, we studied the gender effect on OS and DFS; the results showed no significant difference for either OS (*p* = 0.57) or DFS (*p* = 0.37). Nevertheless, female patients showed better OS and DFS (Figures 3E,F). WBC findings for response-related survival showed that the threshold value (≥50 × 10^9^/L vs. <50 × 10^9^/L) had no significant effect on either OS (*p* = 0.21) or DFS (*p* = 0.30), although patients with WBC (<50 × 10^9^/L) had better OS and DFS (Figures 3G,H and Supplementary Material).

Risk stratification had no significant effect on either OS or DFS. However, the unfavorable group showed poorer OS and DFS (Figures 4A,B). Finally, we studied treatment response-related survival by comparing the CR vs. non-CR patient groups. Our results showed that the CR group had significantly better OS and DFS compared with the non-CR group (*p* < 0.01) (Figure 4C and Supplementary Material).

## Discussion

The current study is the first to be conducted on AML in Qatar from January 2010 until December 2016 for patients aged between 14 and 70 years old. Moreover, it is the first of its kind to determine the cytogenetic abnormalities in AML in adults and to evaluate the risk stratification according to the WHO 2008 classification, and to report the clinical outcomes in Qatar. The median OS rate was around 26.6 months and the median DFS was about 19.5 months. There were no significant differences associated with the prognostic factors (age, gender, WBC, risk stratification, and response to treatment) considered in this study. However, subjects younger than 40 years old, females, and patients with WBC below 50 × 10^9^/L showed better OS and DFS. Age and performance status are the most powerful patient-related risk factors in adult patients with AML (Grimwade et al., 1998; Slovak et al., 2000; Byrd et al., 2002; Döhner et al., 2010; Marchesi et al., 2011). The results showing reduced significance in this study may have been confounded by ethnic variation, a low number of subjects, and the high proportion of patients traveling at different phases of the treatments. Cytogenetics and molecular genetics are considered the most powerful prognostic factors to predict clinical outcomes in AML patients. Furthermore, when they are integrated with WHO classification of AML, they may have a significant impact on patients’ outcomes, which led to the AML risk stratification (Grimwade et al., 1998; Slovak et al., 2000; Byrd et al., 2002; Döhner et al., 2010; De Kouchkovsky and Abdul-Hay, 2016). The most common abnormalities are dominated by t(8;21) in 8.6% of cases and inversion 16/t (16;16) in 7.8% of cases, based on some Western studies (Grimwade et al., 19982010; Slovak et al., 2000; Byrd et al., 2002). These abnormalities are seen in AML *de novo* and are correlated with good prognosis. Trisomy 8, the third abnormality, representing 5.5%, was the most common numerical aberration in our cohort. The rate is 6% in Western countries (Grimwade et al., 1998; Slovak et al., 2000; Byrd et al., 2002), 3% in Malaysia (Meng et al., 2013) and 3.8% in China (Cheng et al., 2009).

The fourth type of abnormality, related to 11q23, was associated with *de novo* AML in our study (Grimwade et al., 1998; Slovak et al., 2000; Byrd et al., 2002). It occurs in no more than 4% of adult AML patients and is correlated with a poor prognosis (Grimwade et al., 1998, 2010; Slovak et al., 2000; Byrd et al., 2002). The frequency of partial and/or complete deletion of chromosomes 5 and 7, which is associated with poor prognosis, ranged from 0 to 2.3% (Grimwade et al., 1998; Slovak et al., 2000; Byrd et al., 2002; Marchesi et al., 2011), significantly lower than the previously reported range of 6–10% in *de novo* AML (Grimwade et al., 1998; Byrd et al., 2002; Cheng et al., 2009). A complex karyotype is found in about 10–12% of AML patients (Marchesi et al., 2011). The incidence of second AML with a poor prognosis increases with age (Appelbaum et al., 2006; Juliusson et al., 2009; Qatar Social Statistics, 2007-2016; Shysh et al., 2018), and the use of leukemogenic drugs is common in such cases (Grimwade et al., 2001; Sanderson et al., 2006; Mrózek, 2008; Haferlach et al., 2012). In routine diagnosis of AML, cytogenetic analysis and WHO 2008 classifications are highly recommended procedures for their central role in the management of the disease (Bacher et al., 2005). Thus, our cohort’s data were analyzed accordingly (Table 4). In fact, 30 AML patients were identified with recurrent genetic abnormalities, 18 with myelodysplasia-related changes, 3 with therapy-related myeloid neoplasms, and 33 not otherwise specified (Table 4).

The results reflect limitations due to the low sensitivity of conventional cytogenetics and the high prevalence of normal cytogenetic AML, as well as a shortage of molecular studies. Failed karyotype and normal karyotype represented 3.2 and 48.4%, respectively.

The missed chromosome aberrations may have been due to technical problems. Trisomy 8 and trisomy 11 have been reported in interphase cells of AML with normal karyotype, probably owing to the inability of the abnormal clone with aneuploidy to proliferate *in vitro* (Frohling et al., 2002). It is difficult to determine the quality of chromosome morphology in the G-banding resolution by a conventional cytogenetic method (Cox et al., 2003). The difficulties also occur in cryptic gene fusions, for example, *NUP98-NSD1*, *CBFA2T3–GLIS2*, and *MNX1–ETV6*, which predict poor outcomes in pediatric and young adult AML (Grimwade et al., 2016). Moreover, t(8;21), carrying a mutation of the *KIT* gene, has a negative impact on outcome, with a significantly lower OS compared with wild-type *KIT* (Klein et al., 2015). Detection of these abnormalities is important to determine the appropriate treatment and decrease the risk of death. Cytogenetic analysis may encounter problems where breakpoints occur in close proximity; for example, at least five different genes that can potentially recombine with the MLL locus fall within the 19p13.1;13.3 regions (Grimwade et al., 2016). Furthermore, cytogenetics provides no clear information regarding the molecular mechanisms underlying AMLs with numerical or other structural changes, or, importantly, those with CN-AML, which account for 40% of adult AML and are highly heterogeneous in terms of clinical outcome (Grimwade et al., 2016).

The ELN classification (2010), recognized by the WHO, divides patients on the basis of CN-AML molecular alterations, namely *NPM1*, *CEBPA*, and *FLT3* mutations (Döhner et al., 2010). Later, in 2016, a new revised version was released. The WHO classification of AML defines six major disease entities based on genetic information together with morphology, immunophenotype, and clinical presentation (De Kouchkovsky and Abdul-Hay, 2016).

Here, we conducted a FISH study of the five most common abnormalities (see section “Patients and Methods”). Such molecular genetic studies have been performed since 2015 and have been applied only to *FLT3* and *NPM1* in CN-AML. The presence of *FLT3*-ITD with wild-type NPM1 predicted a poor prognosis, whereas *NPM1* mutation in the absence of *FL3*-ITD was associated with reduced risk of relapse and improved OS (Döhner et al., 2010; De Kouchkovsky and Abdul-Hay, 2016; Grimwade et al., 2016). Cytogenetic analysis was used to stratify our AML cohort into three groups—low risk (18%), intermediate (57%), and high risk (24%)—concordant with previous reports (Appelbaum et al., 2006; Espirito Santo et al., 2017).

Of the total of 128 patients, 67.7% were in complete remission, 20.5% were resistant to disease, and 11.3% had died by day 30. The CR rate after induction 2 and/or salvage therapy was 81%, and the death rate at day 60 was 9%. The death rate was high owing to infectious disease. The CR rate after inductions 1 and 2 and the resistant disease rate were comparable to those reported by previous studies (Büchner et al., 2012; Burnett et al., 2013; Willemze et al., 2014). The MRC AML15 trial (Burnett et al., 2013) reported a CR rate of 78%, and death rates at days 30 and 60 of 6 and 8%, respectively. The 8-year survival rate was around 72% (favorable 95% and intermediate 63%) in the FLAG–idarubicine arm (two inductions and two consolidations). Moreover, the German AML intergroup study (Büchner et al., 2012) reported a CR rate of 70% in the standard treatment arm; the death rate was 5% in cases of aplasia and 25% in patients with resistant disease. According to the same study, the 5-year survival rate was 44.3% and the 5-year relapse-free survival rate was 44.8%. CR rates were 68.2% after induction 1 and 72% after induction 2. In the EORTC-GIMEMA AML-12 trial (standard treatment arm), the death rate during induction 1 was 9%, the resistant disease rate was 18.9%, DFS at 6 years was 41.6%, and relapse incidence was 47.9% (Willemze et al., 2014). The median survival rate of our cohort was 26.6 months, and the OS at 3 years and 5 years was 40 and 18.3%, respectively. The only predictive prognostic factor affecting survival was response to treatment. The other factors, including age, gender, WBC, and risk stratification, were not statistically significant. However, we noticed a better survival rate in female patients below 40 years old, in patients with WBC less than 50 × 10^9^/L, and in the favorable and intermediate groups.

In our study, the mortality rates at day 30 and day 60 were 11.3 and 9%, respectively, and mainly associated with bacteremia and fungemia. This rate was relatively high when compared to previous studies (Büchner et al., 2012; Burnett et al., 2013; Willemze et al., 2014). However, it falls within acceptable ranges when it is compared to some febrile neutropenia studies (Wisplinghoff et al., 2003; Ruhnke et al., 2014), where the mortality rate is around 36% due to blood stream infections: 18% due to GNB, 13% due to polymicrobial infections, and 5% to GPC.

The high infection incidence in our patients can be attributed to the following reasons. First, the majority of patients are in expatriate services, coming from low-income countries where poor hygienic conditions and GNB invasions are common. Second, in our institution guidelines, the use of antibiotic prophylaxis in AML neutropenic patients is not recommended because it may increase the selection of resistant microbes.

To overcome these problems, since 2017, hematology, Medical Intensive Care Unit (MICU), and the infection disease and infection control teams have been closely collaborating to reduce the incidence of infection in patients with hematological malignancies. In this respect, the following aspects were carefully implemented: (1) national guidelines for febrile neutropenia based on hospital microbial and antibiogram data; (2) antimicrobial stewardship program (ASP) to promote the appropriate use of antimicrobials and help clinicians improve clinical outcomes and minimize harms due to the spread of infections caused by multidrug-resistant organisms; (3) sepsis bundle established and frequently monitored and reviewed to set the best evidence base for maximum care and outcomes for patients; and (4) compulsory detection of carbapenem-resistant organisms through a rectal swab before any chemotherapy for acute leukemia and transplant patients.

In the current study, the stratification of AML was based on conventional cytogenetic analysis as per the WHO 2008 guidelines. The patients received treatment with conventional chemotherapy and/or allogenic transplant. Molecular tools such as reverse transcription-polymerase chain reaction (RT-PCR) and next generation sequencing (NGS) have been included in AML diagnosis since 2017 (Döhner et al., 2017). These techniques are becoming integral part in the initial work-up and follow-up in AML in several hospitals, resulting in target and personalized therapy protocols. However, these techniques are not affordable in many countries, still lack standardization of data analysis, and rely on highly skilled personnel. In the past few years, treatment decisions in AML have become more and more dependent on target therapy. Unfortunately, in NCCCR at Hamad Medical Corporation, molecular testing based on NGS and novel therapies based on *FLT3*, *BCL*-2, and *JAK* inhibitors are not yet available in our setting at the clinical level. Our treatment protocols are still based on conventional cytogenetics and FISH studies.

Novel therapies are showing some promising improvements in AML outcomes (Tamamyan et al., 2017). Target therapy in AML can be categorized in different groups such as: (1) protein kinase inhibitors (PI3K/AKT/mTOR, Aurora and polo-like kinase, CDK4/6, CHK1, WEE1, MPS1 inhibitors, SRC and HCK inhibitors); (2) epigenetic modulators (SGI-110, HDAC, IDH1, IDH2, DOT1L, and BET-bromodomain inhibitors); (3) new chemotherapeutic agents (CPX-351, vosaroxin, nucleoside analogs); (4) mitochondrial inhibitors (Bcl-2, Bcl-xL, Mcl-1, and caseinolytic protease inhibitors); (5) therapies targeting specific oncogenic proteins (fusion transcripts targeting EVI1, NPM1 targeting, and Hedgehog inhibitors); (6) therapeutic and immune checkpoint antibodies [mAbs against CD33, CD44, CD47, CD123, CLEC12A, immunoconjugates (e.g., GO, SGN33A), BiTEs and DARTs, CAR T cells or genetically engineered TCR T cells, immune checkpoint inhibitors (PD-1/PD-L1, CTLA-4), anti-KIR antibody, vaccines (e.g., WT1)]; and (7) cellular immunotherapies and therapies targeting the AML microenvironment (Döhner et al., 2017; Tamamyan et al., 2017). Recent research reported modest achievement in targeted immunotherapies along with curative-intent allogeneic hematopoietic stem cell transplantation in AML (Knaus et al., 2018). The two best-known checkpoints are cytotoxic T-lymphocyte antigen-4 (CTLA-4) (Knaus et al., 2018) and the programmed cell death protein 1 receptor (PD-1) (Giannopoulos, 2019). In AML, increased PD-1 expression on CD8+ T lymphocytes may be a leading factor to immune suppression during the progression course of the disease. CD8+ T cell dysfunction was in part reversible on PD-1 blockade or OX40 costimulation *in vitro* (Daassi et al., 2020). The PD-1 inhibitor nivolumab with HMAs and CTLA-4 inhibitor ipilimumab are still in early phases of clinical trials, and they are commonly associated with immune-related adverse events (irAEs), which can be fatal for patients (Liao et al., 2019). Moreover, it is difficult to consider PD-1 as a prognostic factor in hematological malignancies unless considering how to distinguish between the several forms of soluble and extracellular PDL1 secreted in blood when analyzing responses to immunotherapy (Giannopoulos, 2019).

The main limitations of the study that affected the survival rate and the results in general were the high ethnic diversity among patients, the small number of subjects included in the study, and the missing data due to missing follow-up because many patients traveled during various phases of the treatments. Moreover, postinduction therapy was given to transplanted and non-transplanted patients in the same arm. Implementation of NGS in AML patients’ diagnosis in NCCCR at Hamad Medical Corporation together with protocols for target therapy will be our main focus for better improvement of the quality of care in our institution.

## Data Availability Statement

The raw data supporting the conclusions of this article will be made available by the authors, without undue reservation, to any qualified researcher with respect to institutions standards.

## Ethics Statement

The study was approved by the Medical Research Center Institutional Review Board (MRC-IRB) for the research proposal number “17287/17, 15/5/20”, and was exempted from ethical approval.

## Author Contributions

HEO, RT, AE, AEO, and NK: conceptualization. HEO, AE, AEO, NK, and SE: data curation. HEO, FI, DS, HE, AG, AYE, ZN, and AA: formal analysis. HEO and AEO: funding acquisition and project administration. HEO, RT, AE, NK, HE, AG, FI, DS, SE, ZN, and AA: methodology. HEO, NK, and DS: resources. AEO: software. HEO, RT, and AE: supervision. AEO, ZN, AA, AYE, and AG: validation. AE and AEO: visualization. HEO, AE, RT, and AEO: writing – original draft. HEO, RT, AE, NK, HE, AYE, AG, FI, DS, SE, ZN, AA, and AEO: writing – review and editing.

## Conflict of Interest

The authors declare that the research was conducted in the absence of any commercial or financial relationships that could be construed as a potential conflict of interest.
