# Supplementary material for: Acute Myeloid Leukemia in Qatar (2010–2016): Clinical, Biological, and Prognostic Factors and Treatment Outcomes
Source: Front Genet. 2020 Jun 17;11:553. doi: 10.3389/fgene.2020.00553 (PMC7313235; doi:10.3389/fgene.2020.00553)
Supplement: Supplementary file 1 [file Table_1.DOCX]

**Supplemetary 1:** **Summary of different statistics related to prognostic factors, treatment outcomes, and overall survival in different AML groups per WHO 2008 classification.**
